# Is the Cure Worse than the Disease? The Ethics of Imposing Risk in Public Health

**DOI:** 10.1007/s41649-022-00218-1

**Published:** 2022-09-10

**Authors:** Diego S. Silva, Maxwell J. Smith

**Affiliations:** 1 Sydney Health Ethics, School of Public Health, University of Sydney, Sydney New South Wales, Australia; 2 School of Health Studies, Western University, London Ontario, Canada

**Keywords:** Risk, Relational autonomy, Relational justice, Public health, Complexity

## Abstract

Efforts to improve public health, both in the context of infectious diseases and non-communicable diseases, will often consist of measures that confer risk on some persons to bring about benefits to those same people or others. Still, it is unclear what exactly justifies implementing such measures that impose risk on some people and not others in the context of public health. Herein, we build on existing autonomy-based accounts of ethical risk imposition by arguing that considerations of imposing risk in public health should be centered on a relational autonomy and relational justice approach. Doing so better captures what makes some risk permissible and others not by exploring the importance of power and context in such deliberations. We conclude the paper by applying a relational account of risk imposition in the cases of (a) COVID-19 measures and (b) the regulation of sugar-sweetened beverages to illustrate its explanatory power.

Deliberations about risk and benefit, including the imposition of risk introduced by public health interventions, should be central to whether and how said interventions are designed and implemented; this is the case whether we are reflecting on communicable or non-communicable diseases. Consider:

- The COVID-19 pandemic raises a host of risk-related challenges. First, we need to identify who is at greater and lesser risk of experiencing the various harms associated with the virus, SARS-CoV2, itself. But also, we should consider how the implementation of public health policies to curb the spread of COVID-19 introduce new risks onto people and communities (e.g., stay-at-home orders limit how people who are not internet literate, or do not have ready access to the internet, can order groceries).
- When considering strategies to regulate population-level excess sugar intake, whether it be sugar-sweetened beverages (SSB) or confectioneries, policymakers must balance the desire to promote health with the risk of harms that may come about through measures such as taxation, education, or prohibition (e.g., education campaigns are perhaps less cumbersome than other measures, but they risk not reaching or being as effective with marginalized persons and communities).

In short, examples of risk abound in policies and measures intended to address both communicable and non-communicable diseases. And there are significant implications stemming from exactly how risk is conceptualized in response to these questions and concerns, such as whether, when, and how a public health measure is implemented and who should be targeted or prioritized. The critical observation is that public health interventions, though intended to bring about some good, often carry associated risks that are *imposed* onto persons and communities. In other words, sometimes persons and communities will bear a risk of harm that would not exist but for a public health intervention.

We maintain that understanding how to ethically impose risk can help us reason through difficult cases in public health, specifically those whereby a public health policy or intervention introduces a new risk altogether. As we argue below, evaluating the ethical appropriateness of a public health organization’s measures often requires carefully analyzing who bears the risk of the measure, who benefits from the measure, and the kinds of justifications that are given in defense of the imposition of the measure in the first place. We argue that such risks are imposed via, or in the context of, relationships within and among governments, institutions, businesses, and the public, and hence the permissibility of risk imposition should be informed by *relational* understandings of autonomy and justice. Recent autonomy-based accounts of the ethics of risk imposition tend to use non-relational conceptualizations of autonomy (cf. Cranor 2007; Hansson 2013; Kumar 2015; Oberdiek 2017), while other scholars tend to focus on the just distribution of risks and potential benefits but refer only implicitly to other kinds of justice that are perhaps imperative when evaluating the permissibility or impermissibility of instances of risk (cf. Wolff and de-Shalit 2007). We will try to build on these accounts by arguing that an explicitly relational account of risk imposition can help us better determine and explain when risks imposed via public health measures and actions are justified by bringing into sharper relief the socioeconomic and historical context and complexity of the “real” world in ways that are perhaps absent when not considering relationality. Moreover, clarifying the relational nature of risk should capture the power imbalances that exist when public health interventions are implemented, whether during emergencies or non-emergency times.

We begin by outlining what we mean by “risk imposition” and follow that in the “The Ethics of Risk Imposition to Date — A Selective Summary” section by providing an overview of the thinking that exists about the risk of imposition in the general ethics and public health ethics literature. In the “A Generalized Relational Autonomy Account of Permissible Risk Imposition” section, we outline a generalized account of relational autonomy based on the existing literature and begin to describe what that means for thinking about risk imposition in public health. The “Risk and Relational Justice” section builds off the relational autonomy account of risk imposition, where we defend a relational justice account of risk. Finally, in the “Risk Imposition, Relationality, and Public Health” section, through the examples of COVID-19 and SSB, we show why a relational account of risk imposition better accounts for, and helps us reason through, the justification or not of risk imposition as it relates to public health interventions than non-relational accounts. Due to the realities of word counts, we will not be addressing the question of how to balance the desires or preferences of different autonomous persons as it relates to risk in public health; however, we note that this is a central concern of the theories of risk imposition we will describe throughout. 

## What are “Risk” and “Risk Imposition”?

“Risk” is often understood as a description of a hazard and the probability of the hazard coming about. The notion of a “hazard” suggests a harm, which itself can be described in myriad ways, e.g., Feinberg’s (1988) classic formulation of harm as a setback to interests. For our purposes, the exact nature of harm can be set aside since we are concerned (as will be evident shortly) with the *wrongful* nature of risk, i.e., the wrongfulness of a possible harm given a probability of the harm coming about. “Probability” suggests that we have a sense of the likelihood of something occurring. In public health, as is the case with much of life, the exact likelihood of an event occurring is known with greater or lesser certainty, thus having to account for uncertainty. Finally, part of what makes “risk” a morally difficult issue to navigate is the potential benefit that is conferred to someone by committing the risky action. Like harm, “benefit” has received ample attention in moral philosophy and is understood in myriad ways, including the fulfilment of interests, desires, preferences, and so forth. Again, like harm, the exact definition of benefit can be set aside for our purposes; any intuitive understanding by the reader will suffice.

The moral sense of risk that we wish to interrogate in this paper stems from the idea of *causing* risk and being morally responsible for doing so. In other words, what are the ethics of imposing risk on others? In the last 10 to 20 years, moral philosophers, including bioethicists, have begun to tackle this question in earnest. Jonathan Wolff (2006, 424) provides perhaps the clearest articulation of the challenges associated with causing risk: “…blame attaches itself not to the hazard or the probability but to the cause of the hazard…. Cause concerns how a hazard is created or sustained, and in consequence whether it can be viewed as a matter of culpable human action or inaction”. In other words, for the purposes of normative ethics, it matters who is responsible for causing a risk and how that risk comes about.

For public health ethics, what matters is not just the risk associated with disease, but also the fact that public health measures introduce new risks that would otherwise not exist but for the measure itself. Although perhaps obvious, it bears making explicit that public health measures are commonly enacted in response to pre-existing harms caused by the social, economic, or political marginalization of communities. For example, when we think about the risks associated with the COVID-19 pandemic, we likely first think of the risks of becoming infected with SARS-CoV-2 and the risk of becoming gravely ill or dying thereafter. We also need to account for socioeconomic and political factors that exacerbate the rate and morbidity associated with COVID-19 in certain populations, e.g., Black persons in the USA, persons with disabilities, Aboriginal populations in Australia. Critically, a host of other risks exist because of the public health measures themselves, e.g., loss of income caused by lockdown orders; or depression and anxiety caused by prolonged physical distancing. The risks associated with public health measures are commonly foreseen but unintentionally imposed by governments onto members of the public.^1^ This raises a critical ethics question which the bioethics community has struggled to answer: how do we ethically justify imposing foreseeable risks while choosing to mitigate or avoid others in public health?^2^

## The Ethics of Risk Imposition to Date — A Selective Summary

A basic sense of relationality is straightforwardly entailed by the concept of risk imposition. Outside the category of self-risk, when we talk about the ethics of imposing risks of harm, it is understood that there are at least two agents: the person(s) (or government, corporation, or other agent) who imposes the risk and the person(s) subject to the risk.^3^ In other words, agent *x* stands in relation to agent *y* regarding risk of harm *z*. The field of public health ethics has often implicitly or explicitly addressed the idea of risk of harm, notably via the invocation of the harm principle and the precautionary principle. With regards to the harm principle, JS Mill (2009, 12) is often held as its progenitor, where in *On Liberty* he states: “…the only purpose for which power can be rightfully exercised over any member of a civilized community, against his [sic] will, is to prevent harm to others”. Here, Mill explicitly invokes the idea of prevention of harm, which means the harm has not yet occurred; the harm principle should be, and is, commonly used as a justification for avoiding harm, not rectifying instances where harm has occurred. Moreover, the harm principle is relational in the sense noted at the outset of the paragraph insofar as it has to do with prevention of harm to others and not oneself. Meanwhile, in simple (and perhaps simplistic) terms, the precautionary principle—what some, e.g., Jensen (2002), consider to be a “clarifying amendment” to Mill’s harm principle to aid decision making under circumstances of scientific uncertainty—says that a lack of full scientific certainty should not preclude intervening when there is a threat of serious or irreversible harm (United Nations 1992); here, too, the threats in mind contain at least two agents or groups of agents. Thus, in an important sense, the concept of risk imposition, and its relational nature, forms a foundational part of public health ethics, even if it is not often explicitly considered as such.

Increasingly, public health ethics has begun to engage with the normative ethics scholarship on the challenges associated with risk imposition (Wolff 2006; Munthe 2011; Littmann and Viens 2015; Rogers et al. 2019; Silva and Smith 2020; Silva 2021; John 2021); yet it is unclear the extent to which the literature incorporates and explicitly considers the relational aspects of risk and risk imposition. Rogers et al. (2019, 234) claim that the challenge of imposing risks “…lies in morally appraising situations in which potential actions have complex and as yet uncertain and/or unknown consequences. These situations are common in healthcare where services and interventions typically have many variants, each of which may have an indeterminate mixture of outcomes at both population and individual levels”. The complexity and uncertainty present in the delivery of healthcare exists in public health, too. Although not necessarily central to our discussions of risk imposition in public health below, acknowledging that public health is a complex system — or a combination of complex systems, perhaps — cannot be ignored, either (Mulvaney-Day and Womack 2009; Silva et al. 2018; Wilson 2021). If the hallmark of systems thinking is accounting for the relationship between the parts of a system, complex systems are ones where these relationships are understood inexactly and, furthermore, where causality between *x* and *y* often can be understood neither proximally nor linearly. Thus, any full account of risk imposition in the context of public health needs to grapple with this complexity. In the “Risk Imposition, Relationality, and Public Health” section, we will gesture to this complexity, but we will be unable to give it its due attention.

Unlike complexity and systems thinking, uncertainty has received a fair amount of attention in various fields of research, including bioethics and public health ethics. For our purposes, articulating the exact nature of uncertainty is unnecessary since any commonly accepted understanding will do for our analysis. What is necessary is to acknowledge that decisions are often made in public health with incomplete knowledge and an evolving understanding of the science, relating either to the risks of a public health threat or the risks involved in the use of a public health intervention. The reasons given in favour or against a course of action will depend on numerous factors, including the social values of a particular community or society. Critically, decisions that carry a risk of harm must also be undertaken based on the best scientific knowledge available at the time the decisions are made. We can only demand that decisions are made on the basis of the best available evidence at the time a decision is executed and that it be executed in good-faith. Hindsight may be used to judge whether a public health measure was correct or successful, but the moral valence of a decision can only be judged rightfully based on the knowledge available to the risk imposer and the person subject to the risk at the time of risking.

Within the philosophical literature, the challenge of justifying the imposition of risk against the wishes of an autonomous person has generally garnered a great deal of attention. Given the concern with liberty and autonomy in public health, public health ethicists should participate in this ongoing debate. However, because the manner in which autonomy is used in the context of public health is often understood relationally (Jennings 2016), which we think is appropriate, we will argue that developing a notion of permissible risk imposition in public health that can coexist with notions of autonomy requires adopting a relational autonomy and relational justice perspective, thereby building on the existing normative ethics literature on risk imposition. A relational autonomy account of risk imposition has important ramifications for considerations of social justice as they apply to ethical risk distribution in the context of public health.

## A Generalized Relational Autonomy Account of Permissible Risk Imposition

It is impossible in our modern lives to live risk-free. In turn, it is equally impossible not to impose some form or level of risk on others in our daily lives (e.g., driving cars). If risk is unavoidable, philosophical debate persists as to why being subject to risk ought to be considered *bad*. Hayenhjelm and Wolff (2001, e31) provide a relatively straightforward set of reasons sufficient for the purposes of this paper: “First, a person may fear, or be anxious, about the risk. Second, they may feel that they need to take costly or difficult precautions to reduce the probability of the hazard occurring. Third, the hazard may have further negative effects, and so they may feel that they need to take steps, again, which may be costly or difficult, to minimize the spread of the hazard”. The key ethics question, then, is: when and what makes imposing risk *wrong*? And why and when is it permissible to impose risk that causes fear or forces a person to change their behaviour? Stated differently, despite whatever harm *x* may exist by being subject to a risk of harm *y*, not all instances of *x* or *y* are wrong; thus, an account must be given of the moral difference between permissible and impermissible risk imposition.

One reason imposing risk might be morally wrong is that it infringes upon someone’s interests, desires, or choices to be placed at risk — i.e., that it infringes a person’s autonomy and choices to not be placed at risk. Whatever other aspects are conceptually necessary to constitute an agent and their autonomy, most would agree that moral agents have interests and desires, and that autonomy consists of having a suitable range of choices available to them. Some scholars, such as John Oberdiek, posit that the notion of autonomy is critical for adjudicating which instances of risk imposition are right and wrong (Oberdiek 2012, 2017). Specifically, Oberdiek appeals to Joseph Raz’s (1986, 398) account of autonomy as his starting point, claiming that “… autonomy requires plotting one’s own life and having a range of acceptable options from which to do so” (Oberdiek, 86), thereby agreeing with Raz’s assertion that “… autonomy is exercised through choice”. If, as noted above, being subject to risks and imposing risks on others is simply part of modern life, then it will be impossible that all options (or choices) will always remain open to us. In other words, any serious account of autonomy will recognize quickly that the autonomies of different agents in a society must coexist in a reciprocal manner; thus, there is no real defense for any agent having an unfettered number and kinds of choices. It follows that our ability to impose risk onto ourselves and others is part of what it means to be autonomous. The inherent reciprocal nature of justified risk imposition is key to Carl Cranor’s (2007) sketch of what a non-consequentialist account of risk imposition might look like. He argues, for example, that in practice, autonomy requires dialoguing with those who are subject to the consequences of our actions, including the creation of risks for/upon others. Some scholars, such as Sven Ove Hansson (2013), do not necessarily center their accounts of ethical risk imposition on the notion of autonomy and its value, but note that consenting to a certain set of risks is not indicative that this set is autonomously chosen, e.g., needing money and thus consenting to work in a mine does not necessarily mean a person has chosen their life circumstances that has led to choosing this occupation in the first place. As Hansson (2013, 119) states succinctly: “[t]hat a person makes a choice under circumstances that she cannot influence does not mean that she consents to these circumstances”. Thus, however else we come to understand what constitutes ethically justifiable risk imposition, it will require finding the balance between different persons and their exercising of autonomy.

Although with Oberdiek, Cranor, and Hansson, we can already begin to note that autonomy is invoked in some relational sense, it is only skeletally so; the invocation of “autonomy” in the literature on risk imposition — although far from atomistic and simplistic — does suggest a more minimal or reserved sense of relationality as noted in the previous section when we spoke about the harm principle or the precautionary principle. It is not enough to agree that autonomy can only meaningfully be understood reciprocally, but rather that autonomy is influenced by so much that lies outside what an individual chooses or desires. In other words, we need to build on Raz’s assertion that “autonomy is exercised through choice” and foreground the ways that autonomy is influenced and shaped, and subsequently, what that means for discussions of risk imposition. This is especially true for public health given the centrality of attention on the social (and political, economic, etc.) determinants of health.

What is needed, then, to adequately apply an autonomy-based account of risk imposition in public health is an acknowledgement that autonomy is relational and an analysis of the implications of this acknowledgement (MacKenzie and Stoljar 2000). It is not our intent in this paper to provide an overview of the various types of relational accounts of autonomy, nor outline the various debates between philosophers (e.g., causal versus constitutive accounts). What we need is a generalized account of relational autonomy for the purposes of adjudicating and explaining morally acceptable risk imposition in public health. Generally speaking, relational autonomy takes seriously (a) the influence of various group identities (e.g., familial relationships, friendships, race or ethnicity, gender) that shape an agent’s sense of self and (b) the social, economic, and political forces that often determine the range of choices from which an autonomous agent can choose. Much has been written both about conceptual and practical challenges associated with the notions of identity and external factors in shaping autonomy. For a general account, it suffices to note that our desires and interests are linked to biological preconditions (e.g., genes) but also to the various groups we find ourselves in at various points in our lives, regardless of whether or not we choose to be part of these groups. Childhood and adolescence provide obvious examples of how our identities are shaped by family and friends; however, group membership and social relationships continue to influence who we are throughout our lives, even (or especially) when we choose the groups with whom we will identify. Being part of these groups, which help shape our desires and interests in an evolving and iterative fashion, often provides the justification or explanation for the choices we freely take (or explains the lack of freedom or free choices that may exist in a person’s life). The agent, with their co-created sense of identity, must then make choices given the options that are available to them. Choosing is dependent on the range and quality of choices available; but moreover, and critically, that range and quality of choices are determined by our social, economic, and political realities.

The explanatory power of accounts of relational autonomy in public health, even in generalized forms, is their ability to uphold the moral importance of autonomy while recognizing the ways it is shaped by various conditions outside the agent’s control. This coheres with the vast empirical evidence on the social determinants of health and one’s ability (or inability or difficulty) in choosing to be healthy. Normatively, it helps us make sense of autonomy in the context of both distributive and relational justice (as we will argue in the next section). It calls on those who maintain that autonomy is intrinsically valuable, or those who believe that it is paramount for well-being or flourishing, to pay close attention to the opportunities to be autonomous in the first place, and the manner in which they have been and continue to be unequally distributed. As Baylis et al. (2008, 202) note, “[r]elational theory helps us to appreciate how things get even more complicated as we attend to features of social justice and take seriously the fact that we are not all equally situated with respect to the opportunities we encounter to develop our autonomy skills and pursue our preferences”. In other words, identity and justice are bidirectionally influential on autonomy, insofar as access or a lack of access to a suitable range of choices (whatever that may be) affects our ability to exercise features of our identity through the fulfilment of preferences, desires, and interests, and vice versa. Relational accounts of autonomy allow us to take seriously history and context, including instances of power imbalances between agents, and their subsequent impact on a person’s autonomy.

It is important to recognize that this generalized account of relational autonomy will be amenable to scholars from various schools of thought. In bioethics and in public health ethics, we often associate and correctly exalt the contribution of feminist philosophers on matters of relational autonomy (MacKenzie and Stoljar 2000; Baylis et al. 2008). In addition to feminist accounts, the basic tenets of relational autonomy — particularly as they relate to the just or unjust impact of social, economic, and political factors on choice architecture — are found elsewhere, too. For example, Jennings (2016) argues that republican accounts of liberty as non-domination are dependent on the notion of relationality, as is the deployment of autonomy in capabilities and functionings theories of justice. Moreover, Marxist accounts of freedom (though not the same as autonomy, to be sure) unsurprisingly pay close attention to the socioeconomic factors that shape our choices, e.g., Cohen’s (2011) argument that under capitalism the means of production are such that one person’s freedom is contingent on another person or group of people being unfree. Even classical accounts of liberalism, which are often held as antagonists to the very notion of relational autonomy, have been reinterpreted — we believe correctly, though we will not defend it here — as taking seriously group identity as a necessary condition for autonomy, e.g., Appiah’s (2007, 20) contention that under any serious reading of JS Mill’s or Immanuel Kant’s account of liberalism, “… individuality presupposes sociability, not just a grudging respect for the individuality of others”.^4^ The point is that the basic aspects of relational autonomy, under a general account, can appeal to, or be justified by, a variety of prominent theories. Although confluence of viewpoints does not ensure soundness, it does suggest that any meaningful defense of an autonomy account of risk imposition should be relational in political contexts, including that of public health.

To reiterate, autonomy is central to the ethics of risk imposition, and ethically permissible impositions of risk in the public sphere are those informed by a relational account of autonomy. The types of risks, and how often one is subject to risk, in public health will be influenced greatly by social, economic, and political factors, while one’s identity and group membership will influence whether a risk is taken up (assuming a person can choose to accept a particular health risk). Examples abound, but for the sake of illustrating our point, imagine the economically poor migrants who work as miners in South Africa and are at increased risk of contracting silicosis and tuberculosis because of the nature of the work and their close living quarters (Stoddard and Aruo 2018). In this example, the economic opportunities that a miner has prior to migrating to South Africa for work are limited due to the history of colonialism and exploitation in the southern African region. Their pre-existing social condition makes them more likely to take on the health risks associated with mining given the lack of access to other well-paying jobs due to historical injustices. Their willingness to bear the risk of mining is usually done so to be paid in order to then send home some of their income to their families. The vast majority of these miners are men, so perhaps one can presume they hold a belief that the men must be the primary bread winners for their families. Already we can begin to see how a miner’s sense of identity (e.g., as a man) and group membership (e.g., as a family’s primary economic contributor) will shape his thinking about his moral obligations to others. The range of choices a miner has are limited due to historical circumstance, so they will be predisposed to take on risks to their health for a high-paying job, relatively speaking. Their ability to fight for safety measures in the mines are also curtailed by a number of factors, including how “disposable” they are perceived to be from the perspective of international mining companies working in South Africa (Cohen’s observations ring true here). In such a situation, it becomes increasingly difficult to see how such a lack of opportunities to which the miner is subjected is morally defensible. In other words, what makes the miner in South Africa’s risk acceptance, as imposed by overlapping forces, morally troubling is not that his options are limited, since they are actually increased by having the option to be a miner in the first place; rather, it is the impingement of their autonomy understood relationally that is morally problematic (or particularly so).

## Risk and Relational Justice

A generalized relational account of autonomy can help inform the ethical permissibility of risk imposition, but it is insufficient. This is because even if risk imposition is guided by an account of generalized relational autonomy, risk may still disproportionately negatively affect some populations, as in the case of the miners above. Consequently, ethical risk imposition in public health must also be guided by an account of justice (Wolff et al. 2020).

Most modern theories of justice argue, as a starting point, that all persons are of equal moral worth. In practice, this translates into a fundamental disposition to take everyone’s interests into account equally when distributing social, economic, and political benefits and burdens, instantiated in the various theories of distributive justice in political philosophy. Despite important disagreements about the substantive aims of justice, in the context of public and global health, most accounts agree that justice requires that all people have their basic needs for daily living met, including access to housing, nutritious food, water, and healthcare (Powers and Faden 2006; Venkatapuram 2011; Daniels 2012).^.^

Critically, not everyone can provide for themselves for a multitude of reasons, e.g., poverty, war, lack of stable employment opportunities, and so forth. As noted above, the reasons persons cannot support themselves and their families are often largely outside of their control. These reasons are often complex and overlapping; this empirical claim is central to the arguments put forward by public health researchers who focus on the social determinants of health (Marmot 2005). For decades now, it has been well understood that a person’s health is shaped by factors such as where they were born and the economic policies of governments both local and international. Addressing the social inequalities related to health are a key concern for all aspects of public health.

Returning to the topic of risk and risk imposition, what matters from the viewpoint of justice, particularly distributive justice, is the distribution of risk itself. It is decidedly unjust if it is always the same people or groups who bear the burden of activities that are risky to health (Wolff and de-Shalit 2007). As noted above, in morally troubling instances of risk imposition, persons often undertake activities that pose risks to their health due to their low socioeconomic status or as a result of other social determinants (e.g., racism, sexism). These situations of personal or community risk are unjust, in part, because they are often borne by those who are socially or politically marginalized for the benefit of those persons or groups who are in power or empowered.

Yet, considerations of justice that fall solely within what Iris Marion Young (1990) calls the “distributive paradigm” will often fail to appreciate the relational or interpersonal dimensions of justice that will be critical to assess and address in an account of ethical risk imposition. Relational justice focuses justice concerns largely on the informal and formal treatment of persons through social interaction (Young 1990; Anderson 1999). Whereas distributive justice concerns itself with a desirable distributive pattern requiring us to aim towards realizing that pattern, relational justice enjoins us to treat individuals in accordance with principles that express just relations (e.g., relations of social equality). Only when principles of just relations are satisfied can the distributions resulting from those relations (e.g., of risk) be considered just. As Anderson (2010) argues, “[t]he justice of distributions is derived from an independent standard of the justice of agents, which involves conformity to principles of justice that regulate their conduct”.

Relational justice therefore seeks to situate justice in its social and historical context (Young 1990). For example, justice does not only require the consideration of how risks are distributed; it also (or solely, depending on the account) finds criteria of justice in how individuals (including institutions and the state) are related to the conditions in which people find themselves (Pogge 2004). In other words, relational justice adds a place “…for those who have or share moral responsibility for the justice or injustice” (Pogge 2004, 142). For example, if government policies or measures predating a pandemic contributed to the social or economic disadvantage of some populations (e.g., homeless populations, Indigenous populations, migrant workers), and this disadvantage is likely to be compounded when risks associated with measures to overcome a pandemic are imposed on those populations, a relational account of justice can capture the special moral responsibilities that the government has to prevent this compounded disadvantage when distributing risks.

Relational justice shifts the attention of distributive justice towards the critical investigation of social phenomena like domination, subordination, exploitation, oppression, and marginalization (Young 1990). Because risks are not equally distributed across society and tend to be disproportionately higher among populations disadvantaged by poverty, racism, colonialism, stigma, and other forms of oppression, it is critical that the ethics of risk imposition attend to these relational forms of injustice.

## Risk Imposition, Relationality, and Public Health

We conclude by applying the preceding observations and arguments about risk imposition in public health in two disparate cases, COVID-19 and excess sugar intake via sugar-sweetened beverages (SSB), for the purposes of illustration. What we hope to show is that a generalized account of relational autonomy, coupled with ideas about relational justice, can help capture what is wrong about certain risk impositions in public health and thereby begin to direct us toward what justifies risk imposition.

### Ethical Risk Imposition and COVID-19

Recall that in the section two, we noted that people are subject to — at least — three distinct kinds of risks in the context of the COVID-19 pandemic: first, there are biological factors that increase the risk of morbidity and mortality should one become infected (e.g., being immunocompromised).^5^ Second, certain marginalized groups are at greater risk of infection and progression to disease due to poor pre-existing health, employment, living conditions, and lack of material welfare because of various social, economic, and political factors (e.g., Black or Lantinx persons due to history of racism in the USA). Third, the decisions undertaken by governments and public health officials have imposed, and will continue to impose, additional risks of harm that exist only because measures are undertaken to arrest the spread of COVID-19 (e.g., physical isolation and distancing leading to increases in mental illnesses), which tend to disproportionately harm those who are already marginalized. These three kinds of risks are in part created by, and subsequently shape, an agent’s self-identity and the kinds of choices they are able to make. It is also these second and third kinds of risk that require a moral evaluation of causality — as noted by Wolff above — and that can help us determine the permissibility of that risk imposed by public health measures.

Imagine that due to a lack of social supports before and during the pandemic, a young Black American named Pat concludes, as part of being a member in their community or neighbourhood, that the right thing to do is to help provide their elderly neighbours with groceries. During the height of the pandemic, Pat risks being a victim and vector in the spread of SARS-CoV-2, and thus unintentionally imposes risks on others, but reasons that this is less dangerous than allowing a person at a much higher risk of infection and disease to either procure their own groceries or go without food. The choices presented to Pat are wrong or unjust, yet they exist because of the existing social determinants of ill health, e.g., racism (Yaya et al. 2020; Tsai 2021). The choice ultimately taken by Pat is done so on the basis of their self-identity that exists, in part, relative to the relational injustice they face, which then limits their range of choices; it is likely that such a person would prefer to avoid the risk of becoming infected and then spreading the virus, but feel compelled to do so because of the pre-existing socioeconomic conditions and racism they and their neighbours experience.

The reality of such a situation is undoubtedly more complex in real life, yet it suffices to illustrate the manner in which a generalized relational autonomy and relational justice account of risk imposition can articulate the moral wrongness of Pat’s situation in a way that a non-relational account of risk imposition perhaps cannot. A non-relational account would seem to limit itself to merely consider the actions imposed by governments because of the pandemic, or the actions chosen by an autonomous person in light of proximally imposed public health measures, who then imposes risks onto other autonomous persons, all in a decontextualized fashion. As such, perhaps someone might argue that governments are not responsible for the spread of SARS-CoV-2 should Pat infect others when delivering groceries because they are merely responding to a virus they cannot control. Not so for a generalized relational account of risk: it is precisely the context that matters morally in adjudicating the actions of governments and citizens in response to COVID-19. Under a relational account of risk, the actions of government and society prior to the pandemic shape their causal and moral responsibility for the actions that Pat feels they must take, and in turn would seem to mitigate the blameworthiness of Pat should they infect their neighbours. It is possible that the non-relational and general relational account of autonomy and justice reach the same conclusion regarding the permissibility of the protagonist’s actions; still, if this is true, the relational account has greater moral explanatory power in this and similar examples.

### Ethical Risk Imposition and Sugar-Sweetened Beverages

SSB (i.e., soda, pop, or soft drinks) are a leading source of excess sugar intake (understood as approximately 25–35 g of added sugar intake by an adult per day), which is associated with an increased risk of disease and morbidity, including diabetes and some types of cancer (AHA 2021). While rates of SSB consumption appear to be declining in some high-income countries, they are increasing in low- and middle-income countries (Nutrition Source 2021). Even within high-income countries, persons of lower socioeconomic status tend to drink more SSB than those of higher socioeconomic status. From a public health standpoint, there are several concerns, including the increasingly inequitable effects associated with SSB and the difficulties that exist in trying to stem the rate of SSB consumption. There is clear evidence of food lobbyists’ attempt to undermine the science that associates SSB with non-communicable diseases (Zenone et al. 2021). Moreover, the amount of money spent on various forms of advertising by the SSB industry overshadows what governments can reasonably spend on public health. For example, while the annual operating budget of the WHO is $5.8billion USD in 2021, (WHO 2021) the *advertising* budget alone of Coca-Cola was $4.24billion USD in 2019, while Pepsi’s annual advertising budget is in excess of $4billion USD (Investopedia 2021). Therefore, a local or state public health authority trying to counter the ill effects of SSB will require confronting the SSB industry’s efforts and financial influence.

A public health authority’s attempts to curb the amount of SSB consumed by a population should acknowledge the complexities associated with the broader social aspects of food and drink. For example, research suggests that sharing food and drink, including those that are high in “empty” calories, often signify care and affection between family members who are immigrants from Latin America living in the US or Canada (Mulvaney-Day and Womack 2009). A parent being able to purchase a soft drink for their children might be (relatively speaking) an economically accessible treat compared to healthier options if they are working-class Latinx living in an expensive city like Chicago or Toronto. Advertising by SSB producers function to exploit exactly these facts to sell their products. So, public health is tasked with navigating this complexity in their attempts to reduce SSB consumption. Measures such as increasing taxes at the point of purchase or reducing the size of takeaway cups risks creating new barriers to small but readily obtainable pleasures for Latinx immigrants of lower socioeconomic status. It might be that, on balance, such public health measures are necessary to reduce SSB consumption, but the point is they are not free of potentially imposing some harms onto person and communities.

A relational account of autonomy and justice can help discern what is so morally disturbing about the advertisement of SSB, namely that it exploits individual and group behaviour to sell a product that will contribute to increased rates of some diseases. Stated differently, a particularly troubling aspect of SSB advertising is not just that it creates a risk by introducing a beverage option (thereby increasing choice) that is potentially unhealthy, it is that it intentionally targets the social bonds created by food and drink, particularly within communities that might lack the means of procuring healthier drinks or treats. Likewise for public health, the key moral quandary is not that its measures remove choices, thus impinging on a person’s purported autonomy, but rather (or more morally importantly), it is that an uncritical introduction of such measures risks ignoring the manner in which food and drink shape identity, thereby potentially further marginalizing already marginalized populations (Silva et al. 2013). An account of ethical risk imposition that centres its analysis solely or primarily on the expansion or contraction of choices is likely unable to account for instances where the introduction of risks through public health interventions are morally problematic because of historical, economic, and social reasons. Similar to the example of COVID-19 above, it is conceivable that a government could claim they are not morally responsible for any unfortunate ills that arise from public health interventions  intended to curb sugar consumption. Under a non-relational account, perhaps such an argument might hold, especially if broader social context can be excluded from consideration. However, under a relational account, what governments do or do not do to regulate the SSB industry — thereby targeting upstream causes — could still render it morally responsible for risks of hardships that arrive should public health units, which are extensions of the government, impose measures to curb consumption at the community and individual levels. A relational account of risk is better placed to make such criticisms because of its focus on power, history, and context.

## Conclusion

Autonomy, however conceived, is but a part of the broader theorizing about the ethics of risk imposition, but an important part. The rightness, wrongness, or permissibility of risk imposition turns on the idea that everyone must co-exist as autonomous persons in complex social environments where avoidance of risk is impossible. The claim we have defended herein has been that thinking about risk imposition in the context of public health would benefit from generalized accounts of relational autonomy and relational justice. Taking relationality seriously allows us to better account for the context of a situation and provides greater explanatory power about what makes imposing risk right or wrong in public health.

## Footnotes

1 We will not couch our argument about the potentially bad consequences of risk in terms of the intention/foresight distinction and debate. We note that the bad consequences are merely foreseen to avoid discussions of malice or recklessness, which are important aspects of the debate regarding risk imposition, especially in debates within tort law. It is possible — thought perhaps improbable — that some public health units in some country or other act maliciously when instituting a public health measure that imposes risk onto others, but we can safely assume that most public health officials do not act in such a manner.

2 We are also not investigating whether there is a morally salient distinction between doing or allowing (or action or inaction).

3 Someone, *x*, who imposes risk onto *y* may, at the same time, impose risk onto themselves, *x*, too. The imposition of risk onto oneself requires a different set of considerations, whether or not the self-risk places another person risk, too. We set aside considerations of self-risk as they take us too far afield.

4 One could argue that the generalized account of relational autonomy, as describe herein, is commonplace to many interpretations of liberal theories, and as such, we do not properly distinguish our account of relational autonomy from other accounts that do not bear the moniker. We are sympathetic to this view, hence our referencing of Appiah on this point. That said, though we will not argue for it here, it does seem true that many of the invocations of liberalism, and liberty in particular, in bioethics and public health ethics, tend to be overly and unrealistically individualistic, or downplay the role of relationality; as such, it does seem worthwhile to highlight the relational aspects of autonomy or liberty in the context of public health.

5 We happily concede that what may seem like ‘pure’ or objective biological factors are, themselves, distally caused by social determinants of health, e.g., one may be immunocompromised because they are undergoing chemotherapy for lung cancer caused by years of smoking, which in turn was the result of their poor economic situation.
